# Ganciclovir-induced mutations are present in a diverse spectrum of post-transplant malignancies

**DOI:** 10.1186/s13073-022-01131-w

**Published:** 2022-10-31

**Authors:** Hu Fang, Helen H. N. Yan, Rebecca A. Bilardi, Christoffer Flensburg, Haocheng Yang, Jayne A. Barbour, Hoi Cheong Siu, Michelle Turski, Edward Chew, Zhen Xu, Siu T. Lam, Rakesh Sharma, Mengya Xu, Junshi Li, Ho W. Ip, Carol Y. M. Cheung, Michael S. Y. Huen, E. Alejandro Sweet-Cordero, Ian J. Majewski, Suet Y. Leung, Jason W. H. Wong

**Affiliations:** 1 School of Biomedical Sciences, Li Ka Shing Faculty of Medicine, The University of Hong Kong, Hong Kong SAR, China; 2 Department of Research & Development, South China Hospital, Health Science Center, Shenzhen University, Shenzhen, 518116 China; 3 Department of Pathology, School of Clinical Medicine, Li Ka Shing Faculty of Medicine, The University of Hong Kong, Hong Kong SAR, China; 4 Centre for Oncology and Immunology,, Hong Kong Science Park, Hong Kong SAR, China; 5 The Walter and Eliza Hall Institute of Medical Research, 1G Royal Parade, Parkville, VIC 3052 Australia; 6 Department of Medical Biology, University of Melbourne, 1G Royal Parade, Parkville, VIC 3052 Australia; 7 Division of Hematology/Oncology, Department of Pediatrics, University of California San Francisco, San Francisco, CA USA; 8 Centre for PanorOmic Sciences, Li Ka Shing Faculty of Medicine, The University of Hong Kong, Hong Kong SAR, China; 9 Department of Pathology, Queen Mary Hospital, Hong Kong SAR, China; 10 Department of Medicine, Queen Mary Hospital, Hong Kong SAR, China; 11 The Jockey Club Centre for Clinical Innovation and Discovery, LKS Faculty of Medicine, The University of Hong Kong, Pokfulam, Hong Kong SAR, China

**Keywords:** Ganciclovir, Mutagenesis, Mutational signature, DNA damage, Organ transplant

## Abstract

**Background:**

Ganciclovir (GCV) is widely used in solid organ and haematopoietic stem cell transplant patients for prophylaxis and treatment of cytomegalovirus. It has long been considered a mutagen and carcinogen. However, the contribution of GCV to cancer incidence and other factors that influence its mutagenicity remains unknown.

**Methods:**

This retrospective cohort study analysed genomics data for 121,771 patients who had undergone targeted sequencing compiled by the Genomics Evidence Neoplasia Information Exchange (GENIE) or Foundation Medicine (FM). A statistical approach was developed to identify patients with GCV-associated mutational signature (GCV^sig^) from targeted sequenced data of tumour samples. Cell line exposure models were further used to quantify mutation burden and DNA damage caused by GCV and other antiviral and immunosuppressive drugs.

**Results:**

Mutational profiles from 22 of 121,771 patient samples in the GENIE and FM cohorts showed evidence of GCV^sig^. A diverse range of cancers was represented. All patients with detailed clinical history available had previously undergone solid organ transplantation and received GCV and mycophenolate treatment. RAS hotspot mutations associated with GCV^sig^ were present in 9 of the 22 samples, with all samples harbouring multiple GCV-associated protein-altering mutations in cancer driver genes. In vitro testing in cell lines showed that elevated DNA damage response and GCV^sig^ are uniquely associated with GCV but not acyclovir, a structurally similar antiviral. Combination treatment of GCV with the immunosuppressant, mycophenolate mofetil (MMF), increased the misincorporation of GCV in genomic DNA and mutations attributed to GCV^sig^ in cell lines and organoids.

**Conclusions:**

In summary, GCV can cause a diverse range of cancers. Its mutagenicity may be potentiated by other therapies, such as mycophenolate, commonly co-prescribed with GCV for post-transplant patients. Further investigation of the optimal use of these drugs could help reduce GCV-associated mutagenesis in post-transplant patients.

**Supplementary Information:**

The online version contains supplementary material available at 10.1186/s13073-022-01131-w.

## Background

Recipients of solid organ or allogeneic haematopoietic stem cell transplants (HSCT) have a higher risk of developing cancer. It has been assumed that long-term immunosuppression and viral infection account for the elevated risk of post-transplant malignancies [1, 2]. Indeed, immunosuppressive drugs such as azathioprine are known to damage DNA and are associated with an increased risk of skin cancer [3]. Furthermore, transplant recipients experience higher rates of infection with oncogenic viruses, such as Epstein-Barr virus and herpesvirus 8, which are major risk factors for post-transplant lymphoproliferative disorders [4] and Kaposi sarcoma [5], respectively. Recently, it was reported that the antiviral ganciclovir (GCV) induces a distinctive mutational signature dominated by CA > AA substitutions [6]. This mutational signature was found in blood progenitor cells of patients after HSCT and in two patients from a survey of 3668 solid whole cancer genomes [6]. Furthermore, we had previously observed two individuals (H015 and WEHI-2) with a shared history of acute myeloid leukaemia, HSCT, and colorectal cancer (CRC) carrying the same CA > AA enriched mutational signature [7, 8]. Both patients had received GCV as part of their treatment following HSCT (Additional file 1: Table S1).

GCV and the prodrug valganciclovir are widely used in solid organ transplant and HSCT patients for prophylaxis and treatment of cytomegalovirus (CMV). This study sought to establish the broader prevalence of GCV-associated cancers by interrogating somatic mutations from 121,771 target panel sequenced cancer patients. Furthermore, since transplant patients are often simultaneously administered a range of other drugs, we used cell line and organoid exposure models to investigate treatments that may influence the penetrance and variability in mutational burden observed in GCV-associated malignancies.

## Methods

### Target panel sequencing cancer patient cohort

A set of 121,771 cancer patient samples with somatic mutation and limited clinical information were obtained from AACR Project Genomics Evidence Neoplasia Information Exchange (GENIE) release 11-public [9] (*n* = 104,264) and FoundationMedicine (FM) [10] (*n* = 17,507). AACR Project GENIE is a publicly accessible database of real-world cancer genomics panel sequencing data assembled from cancer centres worldwide and includes the most common cancer types. The GENIE cohort consisted of a mixture of data from 93 different panels ranging from 6 to 1422 genes (average 544), spanning 6 bases to 9.95 megabases (average 1.06 megabases), with the number of mutations ranging from 0 to 9364 (average 7.938). The FM cohort consists of adult solid tumour samples that underwent genomic profiling on a single uniform platform with 287 genes, spanning 0.83 megabases as part of standard clinical care. The number of mutations in the cohort ranged from 1 to 366 (average 6.787) (see Additional file 1: Table S2 for detailed cohort characteristics). For patients with multiple samples in the database, only the most recent sample, indicated by participant age, was included. This combined dataset is used to identify the presence of GCV-associated signature across different cancer types.

### Accessibility analysis for COSMIC census genes

Somatic mutation data was obtained for a set of recurrently mutated genes from the COSMIC census v77 database [11]. Genes with candidate hotspot mutations were identified by selecting those genes with high variance in mutation counts across the gene. We restricted our analysis to the 50 genes with the highest variance and focused on mutations with at least 100 occurrences in COSMIC. TP53 was removed because the volume of hotspot sites dominated the analysis. We used the mutational signature for GCV (GCV^sig^, derived from H015 [8]) to assess the accessibility of hotspot mutations based on their trimer context. We also assessed accessibility at the level of each driver gene by summing the values across all hotspots in that gene.

### Cell line and organoid treatment and whole-genome sequencing

CRC cell lines H414 and HCT116, murine myeloid cells (*HoxA9-Meis1*-transformed primary cells), and a normal human colon organoid line were used as models for GCV treatment. Clonal H414 and HCT116 cells were seeded in a human plasma-like medium (Gibco, A4899101), for whole-genome sequencing experiments, or Dulbecco’s modified Eagles’ medium, for all other experiments, supplemented with 10% foetal bovine serum 24 h prior to drug treatment. To test cell viability, these cells were treated with GCV (Abcam) or ACV (Abcam) ranging from 0.1 to 1000 μM. After 48-h incubation, the cell viability was assessed by trypan blue exclusion assay. For mutation analysis, cells were treated with 100 μM GCV, 100 μM ACV, and/or 1 μM MMF (Roche) for 48 h. A colony (2–4 cells) was then isolated and expanded, as single cell clones were found to be not viable after drug treatment. Following expansion, DNA was extracted for WGS with 150-bp PE sequencing at 30 × using an Illumina Novaseq sequencer.

Myeloid cell lines were generated by infecting E13.5 C57/BL6 liver cells with pMSCV-HoxA9-IRES-Meis1 (a gift from Guy Sauvageau). These cells were infected with pMIG (GFP +) and single cell cloned by plating in Dulbecco’s modified Eagles’ medium with 20% FBS, 0.3% BactoAgar, and 10 ng/mL mIL-3 (WEHI). Individual colonies were expanded and maintained in IMDM with 10% FBS and 10 ng/mL mIL-3. For growth inhibition experiments, myeloid cell lines were seeded and treated with ACV (Hospira AU) or GCV (Pharmaco AU) ranging from 0.00316 to 100 µM. To assess mutagenicity, clonal cultures were treated with GCV and ACV continuously for 13–15 days, then single cell cloned and expanded in liquid culture. Control samples were either untreated or cultured in vehicle (DMSO). DNA was extracted for WGS with 150-bp PE sequencing at 250–600 M reads per sample using an Illumina Novaseq sequencer.

The normal colon organoid was previously generated from pooled sigmoid organoid derived from a 40-year-old female CRC patient [12]. Briefly, bulk sigmoid organoid was trypsinized into single cells, passed through a cell strainer, and then loaded to a BD Influx™ cell sorter (Biosciences). Single cells were selected based on their physical size and molecular granularity. The sorted cells were serially diluted and seeded in Matrigel for expansion. The clonal organoids were manually picked at 1–2 weeks post-sorting. Fresh medium (advanced DMEM/F12, 1 × GlutaMax, 1 × HEPES, 1 × P/S, 50% Wnt3a, 10% RSPO-1, 10% Noggin, 1xB27, 50 ng/mL EGF, 200 ng/mL FGF10, 1 mM N-acetylcysteine, 1 nM gastrin, 2 µM A83-01) was changed every 2–3 days and organoids were passaged every week. Organoids were treated with 1 µM MMF (Roche), 20 μM GCV (Hainan Poly Pharm Co Ltd), or in combinations (1 µM MMF + 20 μM GCV or 1 µM MMF + 40 μM GCV) continuously for 4–6 weeks. The fresh medium was changed every 2–3 days and organoids were passaged every week. DNA was extracted from the bulk culture as the organoids were found to be not viable as single clones after drug treatment. WGS was performed with 150-bp PE sequencing at 30 × using an Illumina Novaseq sequencer.

The concentration for each cell line treatment is provided in Additional file 1: Table S3. A detailed description of the methods associated with cell viability, DNA damage response, and mass spectrometry assays are provided in Additional file 2: Supplementary Methods.

### Mutation calling from whole-genome sequencing data

For H414 and HCT116 and normal human organoids, raw sequencing reads were aligned to the human (hg38) reference genome using bwa (v0.7.17-r1188) [13]. Mutations were called using MuTect2 (v4.2.5.0) [14] and Strelka2 (v2.9.7) [15] were run in paired normal-tumour mode, where the respective parental clone of each cell line was used as the “normal”, while the drug-treated or control cells were used as “tumour”. For MuTect2, mutation calls required support from 3 or more reads with at least one on each strand. The final set of mutations used for the downstream analysis required that a mutation was annotated as PASS by both MuTect2 and Strelka2 and that the mutation was not shared with any other sample.

For data from the murine cell line, reads were aligned to mm10 with bwa (v0.7.17-r1188) [13], and samples from the same clone were grouped for analysis with superFreq (v1.4.3) [16]. For signature analysis, we used somatic variants with somaticP > 0.5, read depth ≥ 15, VAF ≥ 0.25, and required VAF ≤ 0.05 in all other samples. Bases with at least 15 read depth were classified as callable. The median callable region for the clones was 2.0 Gbp (range 1.7 to 2.3 Gbp). Regions classified as Simple_repeat or Low_complexity by repeatMasker were excluded from the analysis.

### Statistical analysis

**Fig. 1:**
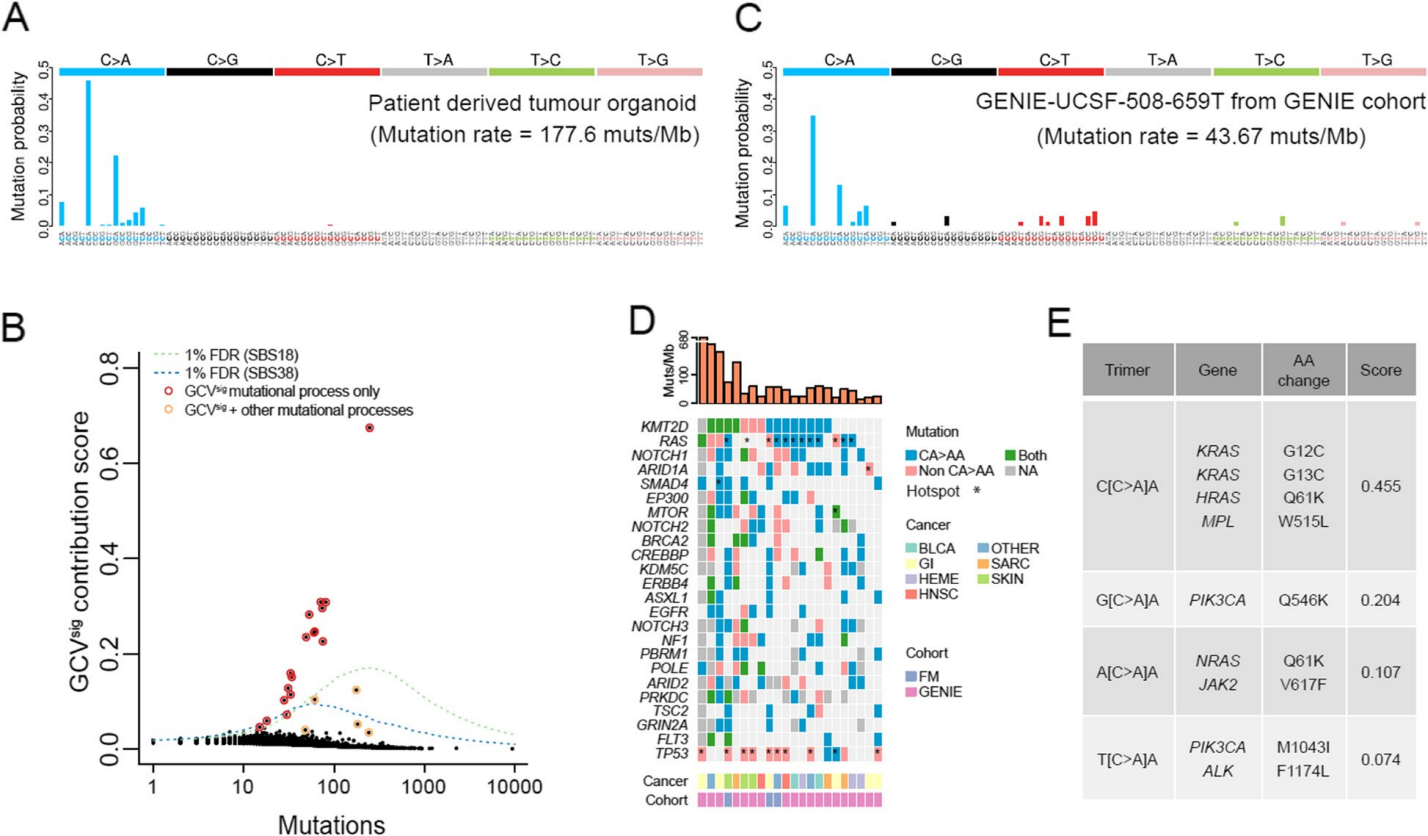
Identification of ganciclovir (GCV)-associated mutational signature from targeted sequencing cancer cohorts. **A** Trinucleotide mutational spectrum from an in-house colorectal cancer patient with GCV^sig^. **B** Identification of samples with GCV^sig^ from patients from AACR Project GENIE and Foundation Medicine cohorts. One percent FDR is set at the sigfit GCV^sig^ contribution score at which there is a 1% chance that the observed mutational spectrum arose from SBS18 (green line) or SBS38 (blue line). Samples with mutation contribution from GCV only or multiple mutational processes are also indicated. **C** Sample spectrum from one example patient from the AACR Project GENIE cohort with GCV^sig^. The mutational spectra of the other 21 GENIE + FM samples with GCV^sig^ are shown in Additional file 3: Fig. S2. **D** Oncoprint of recurrent mutations in CA > AA context across 22 patients with GCV^sig^. Mutations from *KRAS*, *HRAS*, and *NRAS* were combined and labelled as RAS. Cancer type abbreviations: bladder cancers (BLCA), gastrointestinal epithelial cancers (GI), haematolymphoid malignancies (HEME), head and neck cancers (HNSC), sarcoma (SARC), skin cancers (SKIN), and other/unknown primary cancers (OTHER). **E** Mutational potential of known hotspot driver mutations from COSMIC cancer census genes, based on the ability of GCV^sig^ to access trimer sequences (listed in descending order of accessibility)

To identify patients with evidence of GCV-associated mutagenesis, single nucleotide substitutions from each sample were represented based on trinucleotide substitution frequency [17]. The GCV-associated signature (GCV^sig^) was obtained from patient H015 [8] (Fig. 1A). The R package, sigfit [18], was used to identify samples that exhibited GCV^sig^. Briefly, sigfit applies Bayesian inference to fit known mutational signatures to an observed mutational spectrum. Sigfit was used to compute the contribution of mutational signatures (referred to as contribution score) from COSMICv3 with the addition of GCV^sig^ for each patient.

To estimate the false discovery rate (FDR), we simulated trinucleotide mutational spectra based on COSMIC mutational signature SBS18, which shared the highest similarity with GCV^sig^ among common COSMIC mutational signatures (Additional file 1: Table S4). A minor contribution from the age-associated SBS5 was also included in the simulation as a background process. To this end, mutation spectra consisting of 0.95 SBS18 and 0.05 SBS5 were simulated using the R rmultinom function. Sigfit was used to provide an estimate of GCV^sig^ contribution for each simulated mutation spectrum. The process was repeated 1000 times for each mutation count ranging from 1 to 10,000. The 99th percentile GCV^sig^ contribution score from the simulated SBS18 spectra was set as the 1% FDR cutoff for identifying samples with GCV-associated mutagenesis. A GCV^sig^ cutoff was also calculated for SBS38 spectra, but this was only applied to skin malignancies as it has been specifically associated with UV damage [19].

As sigfit assigns uniformly low contribution scores across all signatures when it cannot confidently identify any distinctive mutational processes, samples with less than 10 mutations were excluded as our analysis lacks the ability to confidently detect GCV^sig^ below this mutation count based on our 1% FDR threshold (Additional file 3: Fig. S1A).

While this analysis identified cancers where GCV^sig^ was dominant, we noted that the FDR cutoff based on 0.95 SBS18 contribution would be too stringent for cancers with large contributions from multiple mutational processes. To this end, we further simulated SBS18 and SBS38 spectra at a range of SBS contributions (0.05–0.95, the remainder contribution being SBS5) across a range of mutation counts (5–250) to obtain the 99th percentile GCV^sig^ contribution score from the simulated SBS18 or SBS38 spectra (Additional file 3: Fig. S1B-C). Using these values, we then used the following regression model to estimate the 1% FDR GCV^sig^ score cutoff.

$${{\text{GCV}}^{\text{sig}}}_{\text{ score}}={\beta}_{0}+{\beta}_{1}{X}_{\text{count}}+{\beta}_{2}\mathrm{log}(X_{\mathrm{count}})+{\beta}_{3}{X}_{\mathrm{contrib}}+{\beta}_{4}{X}_{\mathrm{contrib}}\mathrm{log}(X_{\mathrm{count}})$$

where ${X}_{\mathrm{contrib}}$ indicates the SBS contribution fraction and ${X}_{\mathrm{count}}$ is the mutation burden. The model provides a very good estimation of the actual GCV^sig^ score (*R*^2^ = 0.98 and 0.85 for SBS18 and SBS38 respectively, Additional file 3: Fig. S1D-E) and enables the 99th percentile GCV^sig^ cutoff score to be estimated without a computationally costly simulation for each sample. For individual samples with sufficient CA > AA mutations (> 10), the GCV^sig^ contribution is estimated based on the fraction of CA > AA mutations and, along with the total mutation count, is input to the regression model for SBS18 and SBS38 to determine if the observed GCV^sig^ score is above the estimated 99th percentile GCV^sig^ score cutoff. These cutoff values are listed for each sample in Additional file 1: Table S5. To test if the GCV^sig^ of the sample passed this cutoff, the SBS contribution is estimated using the fraction of CA > AA mutations in the sample.

To ensure that our FDR cutoff is robust, particularly for samples with low number of mutations, we further estimated the FDR of GCV^sig^−positive samples for the cohort. To do this, we randomly sampled mutational spectra from the cohort-wide average and calculated the GCV^sig^ contribution score and whether it is above 1% SBS18 or SBS38 FDR as described above. This whole process was repeated 10 times.

Finally, to further account for multiple testing correction, we calculated *p*-values for each sample by comparing the difference between the expected and the observed frequency of mutations attributed to GCV^sig^. This frequency and the frequency of non-GCV^sig^ mutations are calculated respectively as,

$${{{{X}_{\mathrm{GCVsig}}=\mathrm{GCV}}^{\mathrm{sig}}}_{\mathrm{score}}X}_{\mathrm{count}}\ \mathrm{and}\ {{{{X}_{\mathrm{non}-\mathrm{GCVsig}}=(1-\mathrm{GCV}}^{\mathrm{sig}}}_{\mathrm{score}})X}_{\mathrm{count}}$$

Under the null hypothesis, the expected frequency of GCV^sig^ is based on the cohort-wide average mutational spectrum simulated 1000 times. The chi-square test is then used to compute the *p*-value, which is then multiple testing corrected by the Benjamini–Hochberg procedure.

For analysis of significance relating to cell line and organoid models, two-sided Student’s test, ratio *t*-test, or Fisher’s exact test was used as appropriate.

## Results

### Ganciclovir (GCV)-associated mutagenesis is detected across a broad range of cancers

To identify the presence of GCV^sig^ across different cancer types, we analysed genomics data for 121,771 patients who had undergone targeted sequencing compiled by the Genomics Evidence Neoplasia Information Exchange (GENIE) or Foundation Medicine (FM). Despite the relatively small region of the genome covered by targeted sequencing platforms, we were confident that the GCV^sig^ could be detected as the signature is highly distinctive, and we had previously found it associated with hypermutation (> 100 mut/Mb) [8] (Fig. 1A). We identified 22 (0.0181% of 121,771) patients with strong evidence of GCV^sig^ (FDR < 1%, see the “Methods” section) (Fig. 1B, C and Additional file 3: Figs. S2 and S3, Additional file 1: Table S5). After multiple testing correction (see the “Methods” section, Additional file 1: Table S5), 17/22 remained significant (adjusted *p*-val < 0.05), generally with the samples with lower mutations becoming insignificant. Previously, C > A mutations from GCV^sig^ had been described to show replication strand asymmetry with more C > A mutations on the leading strand (or G > T mutations on the lagging strand) [6]. Despite the very low mutation count, 15/22 samples showed more C > A mutations on the leading strand and this bias was also evident in aggregate (Additional file 3: Fig. S4), consistent with the mutations being linked to GCV exposure. Of the 5 samples that were below significance after multiple testing correction, 3 showed leading strand bias; thus, we included all 22 samples for the subsequent analysis.

We were able to obtain detailed clinical information for four samples (of the 22), and all had a history of solid organ transplantation and treatment with valganciclovir, the prodrug of GCV (Additional file 1: Table S1). Of note, the 22 patients spanned a diverse range of malignancies (Fig. 1D), with the highest occurrence in epithelial cancers of diverse organs (*n* = 16), such as the gastrointestinal tract (*n* = 5), skin (*n* = 3), head and neck (*n* = 2), and bladder (*n* = 2); sarcoma (*n* = 3); and haematolymphoid malignancy (*n* = 3), which is consistent with a range of cancers where GCV^sig^ had been recently observed [6–8, 20]. Even though our analysis was only powered to detect GCV^sig^ in samples with 10 or more mutations, those with GCV^sig^ detected all had high mutation burden, typically among the top 1–5% of respective cancer types (Additional file 3: Fig. S5).

To evaluate the potential of GCV-induced damage directly leading to driver mutations, we calculated the ability of the signature to access known drivers from COSMIC census genes. The gene most accessible by GCV^sig^ in our analysis was *KRAS*, which has two hotspot variants in the CCA context. GCV can access multiple hotspot sites in the RAS family, including KRAS (G12C, G13C), HRAS (Q61K), and NRAS (Q61K). Other important hotspots include mutations linked to myeloproliferative disorders, including JAK2 (V617F) and MPL (W515L) (Fig. 1E). This is consistent with the observation that 9 of the 22 patients from the GENIE and FM cohorts have a mutation in one of the *RAS* genes in the CA > AA context (Fig. 1D) and also in line with previous observations in GCV^sig^-associated cancers [6]. Furthermore, all the samples have at least one potential CA > AA protein-altering driver mutation. This suggests that GCV can play a direct role in carcinogenesis.

### GCV but not acyclovir (ACV) induces DNA damage response and GCV^sig^ in cell line models

**Fig. 2:**
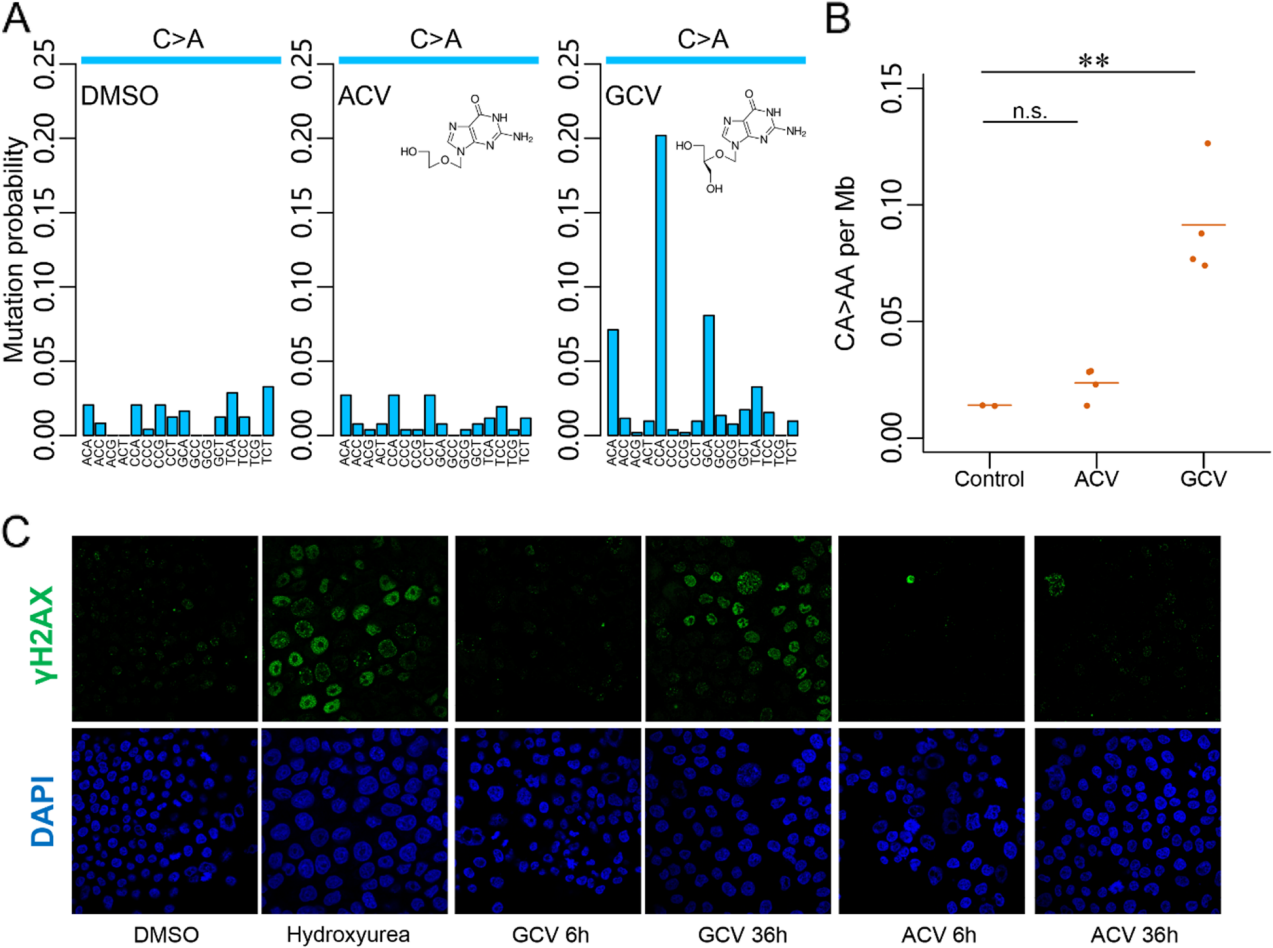
GCV^sig^ and DNA damage response is associated with ganciclovir (GCV) but not acyclovir (ACV) treatment. **A** C > A mutational spectra from whole-genome sequenced (WGS) H414 cell lines treated with vehicle (DMSO), 100 µM ACV, and 100 µM GCV. **B** Contribution of CA > AA mutations across replicates from WGS of control and 5 µM ACV- and 5 µM GCV-treated murine myeloid cells. The mean is shown and statistical significance determined by Student’s *t*-test (** *p* < 0.01, n.s. not significant). **C** 4′,6-Diamidino-2-phenylindole (DAPI) and γ-H2AX staining of H414 cells treated with vehicle (DMSO), 500 µM hydroxyurea, 100 µM GCV, and 100 µM ACV

We reviewed the clinical and drug history of our two patients with HSCT and colorectal neoplasms carrying GCV^sig^ (H015 and WEHI-2) and noted that aside from GCV, they had also received acyclovir (ACV), which could also potentially perturb DNA synthesis [21]. To investigate whether GCV^sig^ is uniquely associated with GCV treatment, we performed WGS of cloned CRC cell line, H414, treated with DMSO, GCV, or ACV. The cell line was generally tolerant of drug treatment (Additional file 3: Fig. S6A) and 100 μM GCV and ACV were selected for the WGS experiments. GCV treatment resulted in the distinctive CA > AA mutational signature, whereas samples treated with ACV showed no such signature (Fig. 2A, Additional file 3: Fig. S6B, Additional file 1: Table S6). To validate the specificity of GCV-associated mutagenesis, treatment and WGS were repeated in murine myeloid cell lines (*HoxA9-Meis1*-transformed primary cells), which were less tolerant of the antivirals than H414 (Additional file 3: Fig. S6C). Despite the substantially lower dose (5 μM), only GCV-treated cells acquired CA > AA mutations compared with ACV and controls (*p* < 0.01, Student’s *t*-test, Fig. 2B, Additional file 1: Table S7).

GCV-triphosphate (GCV-TP) has been reported to be misincorporated into genomic DNA in place of guanosine triphosphate [22]. Consistently, staining of DNA damage response marker γH2AX in H414 showed that accumulation of damage was most evident only after 36 h of GCV treatment when most cells have replicated, while γH2AX signal was largely absent in ACV-treated cells (Fig. 2C). Flow cytometry analysis of the treated cells did not show strong evidence of cell cycle arrest after 36 h of GCV or ACV treatment, supporting the view that H414 cells are generally tolerant of treatment (Additional file 3: Fig. S6D).

### Mycophenolate mofetil treatment potentiates GCV-induced mutagenesis in cells

**Fig. 3:**
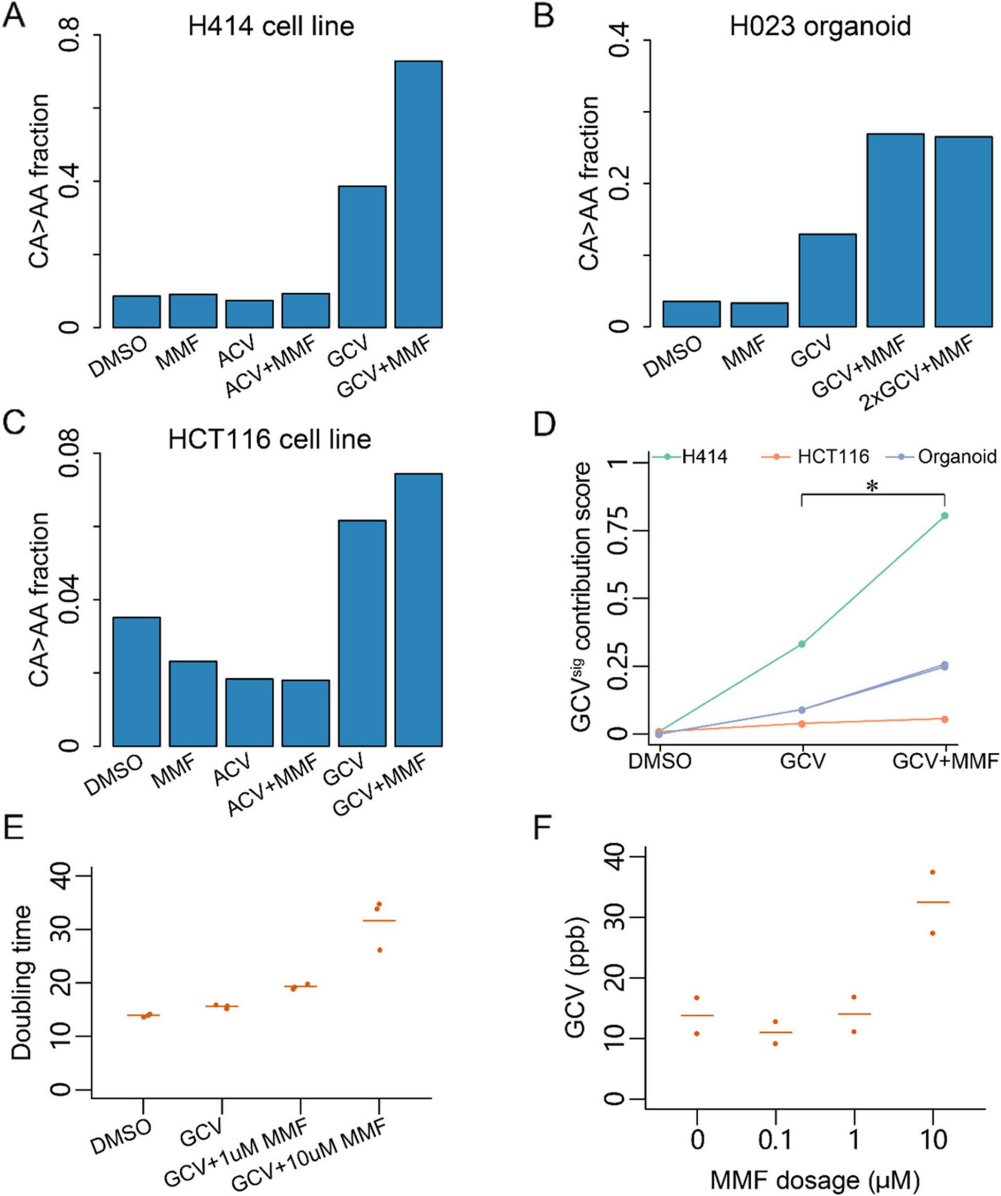
Mycophenolate mofetil (MMF) treatment enhances ganciclovir (GCV)-associated mutagenesis. Fraction of CA > AA mutations in the H414 cell line (**A**), H023 organoid (**B**), and the HCT116 cell line (**C**) treated with vehicle (DMSO), MMF, ACV, ACV + MMF, GCV, or GCV + MMF. The concentration of MMF is 1 µM in all treatments. For H414 and HCT116 cells, the concentration of ACV and GCV is 100 µM. For H023, the concentration of GCV is 20 µM and 40 µM (2 ×). **D** GCV^sig^ contribution score computed using sigfit for the mutational spectra of the treated cell lines and organoids in **A**–**C**. **E** Doubling time of H414 cells under different treatment conditions. GCV concentration was constant at 100 µM. The line indicates the mean of replicates. **F** Liquid chromatograph-tandem mass spectrometry-based quantification of GCV incorporation into genomic DNA under increasing MMF dosage in 100 µM GCV-treated H414 cells. The line indicates the mean of replicates

While GCV is widely used in post-transplant patients, it is unclear what other factors, such as dosage, drug interactions, genetics, or environmental factors, contribute to its penetrance and mutational burden. MMF is an immunosuppressant that disrupts guanosine synthesis and is frequently used in transplant patients [23], including those found with GCV^sig^ from this study and our two patients (Additional file 1: Table S1). To test if MMF might influence GCV-induced mutagenesis, we performed combination drug treatments followed by WGS in H414 cells. The fraction of mutations attributed to CA > AA was higher with combination treatment than GCV alone (*p* < 0.0001, Fisher’s exact test, 0.728 versus 0.387, Fig. 3A, Additional file 3: Fig. S6B, Additional file 1: Table S6). MMF alone did not induce any proportional increase in CA > AA mutations when compared with the DMSO control (*p* = 0.878, Fisher’s exact test, 0.091 versus 0.086, Fig. 3A, Additional file 3: Fig. S6B, Additional file 1: Table S6). The fraction of CA > AA mutations was also higher in organoids treated with MMF and GCV, compared with GCV alone (*p* < 0.0001, Fisher’s exact test, 0.269 versus 0.129, Fig. 3B, Additional file 3: Fig. S7A, Additional file 1: Table S6). In this system, increasing GCV dosage from 20 to 40 µM did not further increase the fraction of CA > AA mutations (*p* = 0.7721, Fisher’s exact test, 0.269 versus 0.265, Fig. 3B, Additional file 3: Fig. S7A, Additional file 1: Table S6).

We further assessed the impact of GCV-induced mutations with and without MMF in cells with DNA mismatch repair deficiency (MMRd). Despite the very high background mutation rate arising from MMRd in HCT116 cells, an increase in the portion of CA > AA mutations could be observed with GCV treatment compared with DMSO control (*p* < 0.0001, Fisher’s exact test, 0.062 versus 0.035, Fig. 3C, Additional file 3: Fig. S7B, Additional file 1: Table S6). This was further increased when used in combination with MMF (*p* = 0.03, Fisher’s exact test, 0.074 versus 0.062, Fig. 3C, Additional file 3: Fig. S7B, Additional file 1: Table S6). Using sigfit, the GCV^sig^ contribution score significantly increased between GCV alone and GCV with MMF across the various treatments in cell lines and organoids (*p* = 0.0134, ratio *t*-test, Fig. 3D).

Although MMF treatment strongly increased the doubling time of H414 cells (*α* = 1.51, *R*^2^ = 0.8869, *p* < 0.001, Fig. 3E, Additional file 3: Fig. S7C), prolonged exposure of cells to GCV prior to cell replication is unlikely to increase mutagenesis as GCV-associated DNA damage occurs during DNA replication [22]. To test whether MMF may have a synergistic effect on GCV-TP misincorporation, we used a mass spectrometry-based assay to quantify GCV in genomic DNA [24] (Additional file 3: Fig. S7D). The level of GCV in DNA was found to increase from 13.8 ppb without MMF to 32.5 ppb at 10 µM MMF (Fig. 3F, Additional file 3: Fig. S7E). This suggests that MMF likely increases the mutagenic potential of GCV by increasing its rate of misincorporation into genomic DNA.

## Discussion

Post-transplant malignancy has typically been thought of as a natural consequence of sustained immunosuppression, but our results point to an active role for the antiviral GCV in causing mutations that may lead to cancer. Cancers with high levels of GCV^sig^ were rare in this group of unselected cancer patients, occurring at approximately 1 in 5500 individuals. This may underestimate the impact of GCV, because of the high level of signal required for detection using a targeted panel. GCV^sig^ is also likely to be far more common in transplant recipients, who regularly receive antiviral therapies. We show that these cancers harbour driver mutations that are accessible by GCV-associated mutagenesis, implicating DNA damage from GCV as a causative factor in initiating the disease. Furthermore, we found that MMF may potentiate the mutagenic effect of GCV in cell line and organoid models.

The finding of GCV-associated mutagenesis across diverse cancer types spanning epithelial, mesenchymal, and haematolymphoid lineages suggests that GCV could exert a broad mutagenic effect on most cells in the body during the course of GCV treatment. In support of this, we previously observed GCV^sig^ not only in the tumour tissue and tumour-derived organoid, but it was also found in an organoid generated from the normal colon of patient H015, albeit at lower levels [8]. Furthermore, GCV^sig^ was observed in normal blood stem and progenitor cells after bone marrow transplantation [6]. As GCV is widely prescribed in post-transplant patients, both as prophylaxis and treatment for CMV, most post-transplant patients are likely to have some level of exposure to the drug. Future studies of somatic mutations in normal cells and tissues from GCV-treated individuals will help establish the prevalence and cell types most vulnerable to GCV-induced mutagenesis. This may ultimately provide important clues in the potential monitoring of genotoxicity of GCV in patients undergoing treatment.

GCV is a guanine analogue that is phosphorylated and misincorporated into genomic DNA [22]. Given that the rate of GCV misincorporation is likely to be influenced by the composition of the nucleotide pool, we hypothesised that MMF, an inhibitor of inosine monophosphate dehydrogenase, might potentiate the mutagenic effect of GCV. Indeed, using cell line and organoid models, MMF was found to increase the incorporation of GCV in genomic DNA and the proportion of CA > AA mutations. The finding has pronounced clinical relevance since MMF is commonly used long-term in solid organ transplant patients as part of a steroid-sparing regime and for treating graft versus host disease in HSCT recipients. Since co-existing CMV infection is common in these patients, the risk of inducing GCV mutations could vary depending on the dosage and duration of drug usage. If the interaction between GCV and MMF is verified in vivo, then the combined long-term use of the two drugs should be cautioned. We note that patient H015, who displayed an extreme level of GCV^sig^ in her CRC and normal colon [8], had concurrently received both drugs for over 5 years prior to colon cancer onset. We did not see elevated mutation rates in response to treatment with ACV, but ACV is much less effective against CMV compared to GCV. Further well-controlled population studies or clinical trials will be needed to document the cancer risk of GCV (and valganciclovir) alone or in combination with mycophenate, and whether other anti-CMV agents may be used instead to limit GCV exposure. For example, foscarnet, which has been shown to be non-mutagenic in cell culture [6], has been shown to have comparable efficacy and toxicity to GCV both as preemptive and first-line CMV treatment [25, 26]. However, foscarnet is administered intravenously, which will limit its use in some instances.

Another interesting observation from our study is that GCV^sig^ is associated with a broad range of transplantation types. Cancers with GCV^sig^ were previously reported in patients who had HSCT or kidney transplants [6]. In the four target panel sequenced patients where we have detailed clinical information, three had received a kidney or kidney and pancreas transplant, and the other had a lung transplant. Meanwhile, our two patients both received HSCT [7, 8]. This suggests that GCV treatment induces mutations across a range of transplant protocols. It would be beneficial to look further at how this relates to CMV status and clinical history to evaluate the level of GCV exposure. CMV infection and the prescription of GCV are generally common in immunocompromised individuals. It would be important to establish whether GCV-associated cancers are also present in other immunocompromised patients, such as those with acquired immunodeficiency syndrome (AIDS) due to infection of human immunodeficiency virus (HIV). As MMF is not part of the standard of care in HIV/AIDS, such a study could help further define interactions between treatments and the conditions under which GCV-associated cancers are more likely to develop.

In our cell line models, we sought to compare GCV^sig^ in MMR-deficient (HCT116) and MMR-proficient (H414) cell lines. We observed that the relative contribution of GCV^sig^ was lower in HCT116 due to increased background C > T and T > C mutations resulting from replication error (Fig. 3A, C). Comparing absolute counts in our data (Additional file 3: Fig. S8) may not reflect differences in the rate of GCV-induced mutation formation as the cancer cells have some level of polyploidy and showed variable growth characteristics, cloning efficiency, and drug response (Additional file 3: Fig. S6A). To control for polyploidy, we evaluated the variant allele frequency of diploid regions (Additional file 3: Figs. S9 and 10) and found that there is still variation of clonality across the samples. Further experiments will need to be conducted in a wider range of MMR-proficient and MMR-deficient cell lines along with confirmation using an in vitro mismatch repair and excision assay to fully evaluate the impact of MMR on GCV-induced mutagenesis [27].

A caveat of using target panel sequencing to detect mutational signatures is that the number of mutations observed is limited compared to whole genomes and exomes. In our analysis, we found that FDR estimation is unreliable for samples with less than 10 mutations. As such, it is possible that our analysis underestimates the true prevalence of GCV-associated mutagenesis. Nevertheless, consistent with our prevalence estimate, we note that there were only two further cancers potentially with GCV^sig^ from a recently published cohort of 12,222 whole cancer genomes [28]. It remains to be established whether GCV^sig^ is more common as a background mutational process in the normal cells of transplant recipients who have been treated with GCV. Another limitation relates to using cell line and organoid models to examine GCV- and MMF-associated mutagenesis. It will be important to establish the contribution of MMF to GCV-induced mutagenesis using patient material or in vivo animal models or through pharmacoepidemiology investigations.

## Conclusions

This study provides further evidence that GCV treatment influences cancer development in diverse organs. While the prevalence is relatively low, at 1 in 5500 in an unselected cohort of 121,771 cancer patients, it is likely much more common in patients who have undergone organ transplantation. Our cell line and organoid models further suggest that co-treatment with MMF increases the mutagenicity of GCV. Further studies should be performed to characterise the safety profile of GCV and combination treatments in humans.

## Supplementary Information

**Additional file 1: Table S1. **Clinical and mutational information of patients with GCV^sig^ identified in this study and from our previous patients. **Table S2.** Patient and panel characteristics of GENIE and FM cohorts. **Table S3**. Concentrations used for the treatment of respective cell lines for WGS mutation experiments. **Table S4. **Cosine similarity of COSMICv3 signatures with GCV^sig^. **Table S5.** GENIE and FM samples analysed in the study with their trinucleotide mutational signature and similarity to GCV^sig^ shown. **Table S6. **Mutational spectrum and sigfit contribution score to COSMIC signatures for H414, HCT116 and normal colon organoid H023. **Table S7. **Mutational spectrum of mouse myeloid cell line.

**Additional file 2. **Supplementary Methods.

**Additional file 3: Figure S1. **Determination of sensitivity and false discovery rate based on SBS18 and SBS38. **Figure S2.** Trinucleotide mutational spectra of the additional 21 samples with GCVsig detected from AACR Project GENIE and Foundation Medicine cohorts. **Figure S3.** Permutation test to determine cohort-wide false discovery rate. **Figure S4**. Replication strand bias for cell lines and cancer samples. **Figure S5.** Mutation burden of GCV positive samples relative to other samples by cancer type. **Figure S6. **Treatment of cells with ganciclovir and acyclovir. **Figure S7. **Treatment of cells with ganciclovir (GCV) and mycophenolate mofetil (MMF). **Figure S8.** Absolute mutations for GCV-induced mutations(CA>AA), C>T and T>C mutations across H414, HCT116 cell line and H023 organoid models. **Figure S9**. Copy number variation across the genomes of H414 and HCT116 clones. The ploidy values are calculated using Control-FREEC from each sample’s whole genome sequencing data. **Figure S10**. Histogram showing the distribution variant allele frequency (VAF) of mutations from diploid regions in each clone of H414 (A) and HCT116 (B).

## Data Availability

Raw sequencing reads are available from NCBI SRA PRJNA830636 (https://www.ncbi.nlm.nih.gov/bioproject/PRJNA830636) [29] for H414 and HCT116 cell lines and PRJNA837717 (https://www.ncbi.nlm.nih.gov/bioproject/PRJNA837717) [30] for murine myeloid cell lines and EGA EGAS00001006707 (https://ega-archive.org/studies/EGAS00001006707) [31] for organoids.

## Abbreviations

GCV: Ganciclovir
MMF: Mycophenolate mofetil
GENIE: Genomics Evidence Neoplasia Information Exchange
FM: Foundation Medicine
HSCT: Haematopoietic stem cell transplants
CRC: Colorectal cancer
CMV: Cytomegalovirus
GCV^sig^: GCV-associated signature
FDR: False discovery rate
GCV-TP: GCV-triphosphate
MMRd: Mismatch repair deficiency
HIV: Human immunodeficiency virus
AIDS: Acquired immunodeficiency syndrome
