# Supplementary material for: Ganciclovir-induced mutations are present in a diverse spectrum of post-transplant malignancies
Source: Genome Med. 2022 Oct 31;14:124. doi: 10.1186/s13073-022-01131-w (PMC9620652; doi:10.1186/s13073-022-01131-w)
Supplement: Supplementary file 2 — Additional file 2. Supplementary Methods. [file 13073_2022_1131_MOESM2_ESM.docx]

**Supplementary Methods**

*Copy number variation and variant allele frequency analysis*

To evaluate the clonality of the cell lines, we first identified the diploid regions for the genomes using Control-FREEC[6] on the whole genome sequencing data. Based on mutations within diploid regions common across all samples, the distribution variant allele frequency of the mutations was summarised in a histogram.

*Replication strand bias analysis*

Left (leading)- and right (lagging)-replicating region annotations was obtained from[7]. For a given mutation type in the right replication direction, the mutation counts (*N_1_*) in that region were calculated, and its complementary mutation was calculated as *n_1_*. Correspondingly, the mutation counts of this mutation type in left replication direction were calculated as *N_2_*, and its complementary mutation was calculated as *n_2_.* The final count of a mutation type for each strand is defined as *N_1_+n_2_* (lagging) and *N_2_+n_1_* (leading).

*Immunofluoresence for DNA damage response*

For Immunofluorescence, cells were seeded into a 6-well plate with coverslips as described and then treated with DMSO, Hydroxyurea (500 μM, Abcam) or GCV (100 μM, Abcam) for 6 hours and 36 hours. Thereafter, cells were washed with PBS and pre-extracted with 0.5% Triton X-100 for 15 seconds followed by fixation with 4% paraformaldehyde for 15 minutes at room temperature. Cells were incubated with γH2AX antibody[8] diluted at 1:200 in 3% bovine serum albumin (BSA) solution for 1 hour at room temperature, followed by PBS washing and 1 hour secondary (Alexa Fluor 488, diluted 1:1000 in 3% BSA) conjugation. Cell nuclei were stained by DAPI and images were captured by Carl Zeiss LSM 880 confocal microscope.

*Cell cycle analysis*

Cells were trypsinized and fixed with drop-wise addition of ice-cold 70% ethanol. After overnight incubation in -20℃, fixed cells were washed once with PBS and were treated with 200 μL of sodium citrate solution containing RNase A for 30 min at room temperature, followed by addition of another 200 μL of sodium citrate solution containing 50 μg/mL propidium iodide (PI). Cell-cycle distribution was determined using Agilent NovoCyte Quanteon analyzer.

*Mass spectrometry analysis of GCV incorporation into genomic DNA*

Cell pellet was extracted by 80% MeOH. Samples were passed through a 10K molecular filter (Merck Millipore). Both the filtrate and residue were retained for further processing. The residue was digested to single nucleotides using a DNA digest mix. To prepare the metabolites for MS analysis, nucleotides were dephosphorylated, then desalting and concentrated using a SPE cartridge (Waters). The sample was then loaded into a LC-MS/MS system (Waters Acquity I-Class and AB Sciex QTRAP®6500+). Detection of GCV was facilitated using the 256.2 m/z 🡪 152.1 m/z transition. Data analysis was performed using the SciEX OS-Q Analysis Software.

**References**

1. Yan HHN, Siu HC, Ho SL, Yue SSK, Gao Y, Tsui WY, Chan D, Chan AS, Wong JWH, Man AHY *et al*: **Organoid cultures of early-onset colorectal cancers reveal distinct and rare genetic profiles**. *Gut* 2020, **69**(12):2165-2179.

2. Li H, Durbin R: **Fast and accurate short read alignment with Burrows-Wheeler transform**. *Bioinformatics* 2009, **25**(14):1754-1760.

3. Benjamin D, Sato T, Cibulskis K, Getz G, Stewart C, Lichtenstein L: **Calling somatic SNVs and indels with Mutect2**. *BioRxiv* 2019:861054.

4. Kim S, Scheffler K, Halpern AL, Bekritsky MA, Noh E, Kallberg M, Chen X, Kim Y, Beyter D, Krusche P *et al*: **Strelka2: fast and accurate calling of germline and somatic variants**. *Nat Methods* 2018, **15**(8):591-594.

5. Flensburg C, Sargeant T, Oshlack A, Majewski IJ: **SuperFreq: Integrated mutation detection and clonal tracking in cancer**. *PLoS Comput Biol* 2020, **16**(2):e1007603.

6. Boeva V, Popova T, Bleakley K, Chiche P, Cappo J, Schleiermacher G, Janoueix-Lerosey I, Delattre O, Barillot E: **Control-FREEC: a tool for assessing copy number and allelic content using next-generation sequencing data**. *Bioinformatics* 2012, **28**(3):423-425.

7. Vohringer H, Van Hoeck A, Cuppen E, Gerstung M: **Learning mutational signatures and their multidimensional genomic properties with TensorSignatures**. *Nat Commun* 2021, **12**(1).

8. Huen MSY, Grant R, Manke I, Minn K, Yu XC, Yaffe MB, Chen JJ: **RNF8 transduces the DNA-damage signal via histone ubiquitylation and checkpoint protein assembly**. *Cell* 2007, **131**(5):901-914.
