# Supplementary material for: Ganciclovir-induced mutations are present in a diverse spectrum of post-transplant malignancies
Source: Genome Med. 2022 Oct 31;14:124. doi: 10.1186/s13073-022-01131-w (PMC9620652; doi:10.1186/s13073-022-01131-w)
Supplement: Supplementary file 3 — Additional file 3: Figure S1. Determination of sensitivity and false discovery rate based on SBS18 and SBS38. Figure S2. Trinucleotide mutational spectra of the additional 21 samples with GCVsig detected from AACR Project GENIE and Foundation Medicine cohorts. Figure S3. Permutation test to determine cohort-wide false discovery rate. Figure S4. Replication strand bias for cell lines and cancer samples. Figure S5. Mutation burden of GCV positive samples relative to other samples by cancer type. Figure S6. Treatment of cells with ganciclovir and acyclovir. Figure S7. Treatment of cells with ganciclovir (GCV) and mycophenolate mofetil (MMF). Figure S8. Absolute mutations for GCV-induced mutations(CA>AA), C>T and T>C mutations across H414, HCT116 cell line and H023 organoid models. Figure S9. Copy number variation across the genomes of H414 and HCT116 clones. The ploidy values are calculated using Control-FREEC from each sample’s whole genome sequencing data. Figure S10. Histogram showing the distribution variant allele frequency (VAF) of mutations from diploid regions in each clone of H414 (A) and HCT116 (B). [file 13073_2022_1131_MOESM3_ESM.docx]

**Figure S1.** Determination of sensitivity and false discovery rate based on SBS18 and SBS38.

**Figure S2.** Trinucleotide mutational spectra of the additional 21 samples with GCV^sig^ detected from AACR Project GENIE and Foundation Medicine cohorts.

**Figure S3.** Permutation test to determine cohort-wide false discovery rate.

**Figure S4**. Replication strand bias for cell lines and cancer samples.

**Figure S5.** Mutation burden of GCV positive samples relative to other samples by cancer type.

**Figure S6.** Treatment of cells with ganciclovir and acyclovir.

**Figure S7.** Treatment of cells with ganciclovir (GCV) and mycophenolate mofetil (MMF).

**Figure S8.** Absolute mutations for GCV-induced mutations(CA>AA), C>T and T>C mutations across H414, HCT116 cell line and H023 organoid models.

**Figure S9**. Copy number variation across the genomes of H414 and HCT116 clones. The ploidy values are calculated using Control-FREEC from each sample’s whole genome sequencing data.

**Figure S10**. Histogram showing the distribution variant allele frequency (VAF) of mutations from diploid regions in each clone of H414 (A) and HCT116 (B).

**
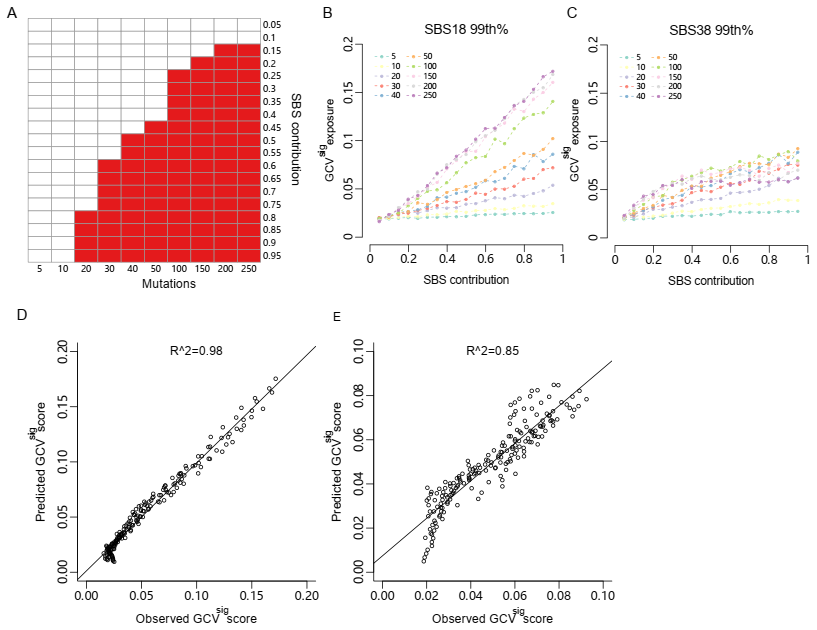
**

**Figure S1. Determination of sensitivity and false discovery rate based on SBS18 and SBS38.** (A) Mutation-signature contributions at which the 5th percentile GCV^sig^ contribution score from a simulated GCVsig mutational spectrum is higher than the 99th percentile GCV^sig^ contribution score from a simulated SBS18 mutational spectrum (denoted in red). Relationship between mutation count and GCV^sig^ contribution score with increasing SBS18 (B) and SBS38 (C) contribution. GCV contribution score is based on the 99th percentile highest score from 1000 simulated SBS18/SBS38 containing mutational spectrum. Correlation between predicted and observed GCV^sig^ score based on 99th percentile GCVsig score from simulated SBS18 (D) and SBS38 (E) mutational spectra with varying signature contribution and mutation burden.


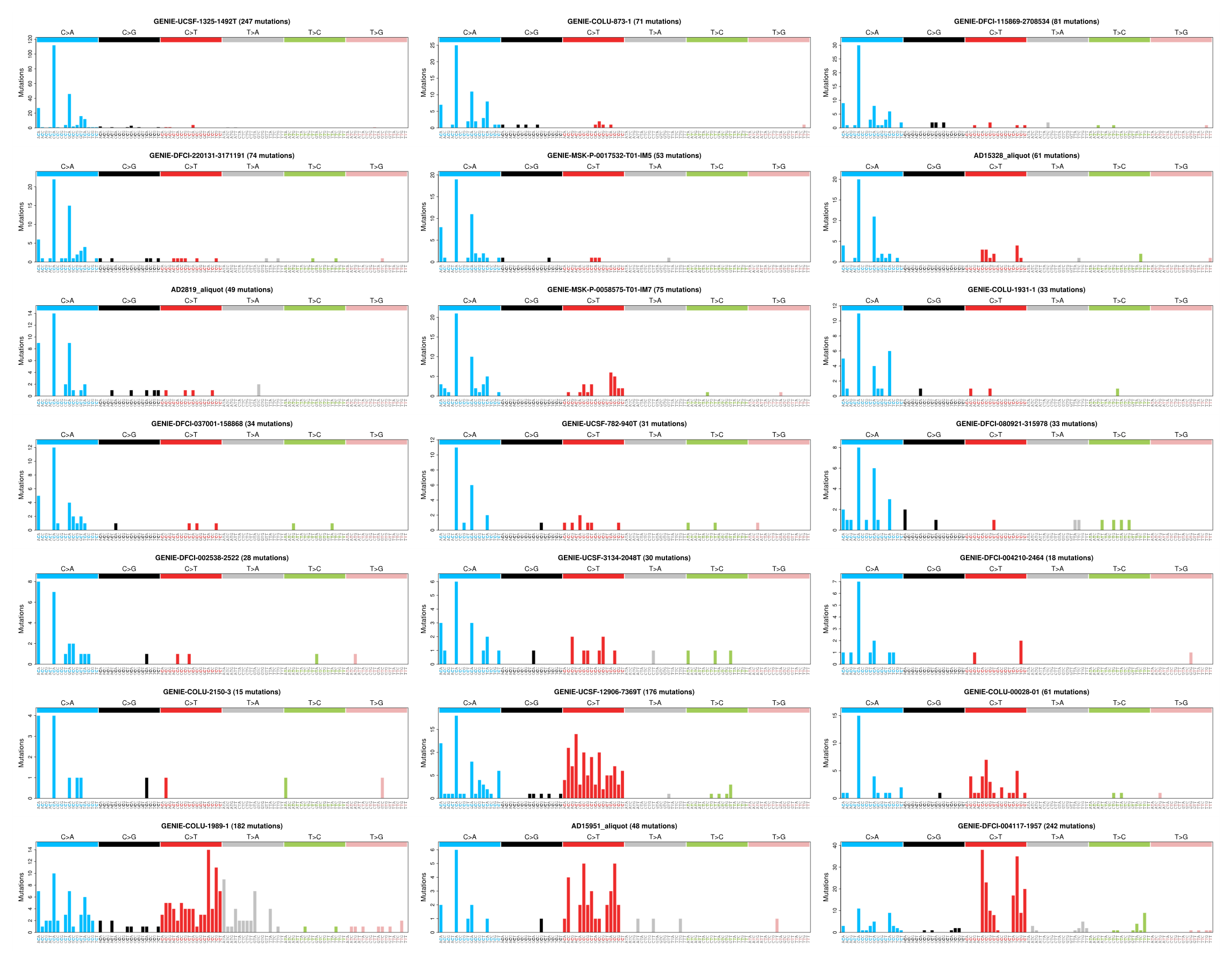


**Figure S2. Trinucleotide mutational spectra of the additional 21 samples with GCV^sig^ detected from AACR Project GENIE and Foundation Medicine cohorts.**


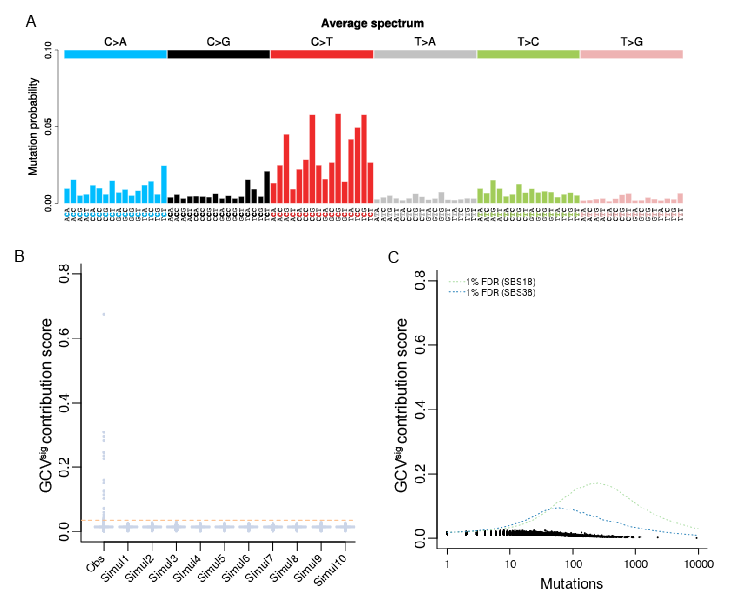


**Figure S3. Permutation test to determine cohort-wide false discovery rate.** (A) Average mutational spectrum across whole GENIE+FM cohort. (B) Distribution of GCV^sig^ contribution scores for the observed (GENIE+FM cohort , n = 121774) in comparison with 10 randomly shuffled cohorts where the mutations of each sample are drawn randomly from the average mutational spectrum. (C) GCV^sig^ contribution from simulated samples. 1% FDR based on SBS18/SBS38 is indicated.

**Figure S4. Replication strand bias for cell lines and cancer samples.** The sample labelled “Combined” is generated using mutations from all GCV^sig^ positive samples combined. Samples with multiple mutational processes are indicated by red colour. Note that the replication strand bias annotation does not span the whole genome, therefore, not all mutations in each sample are included in the evaluation of replication strand bias.

**
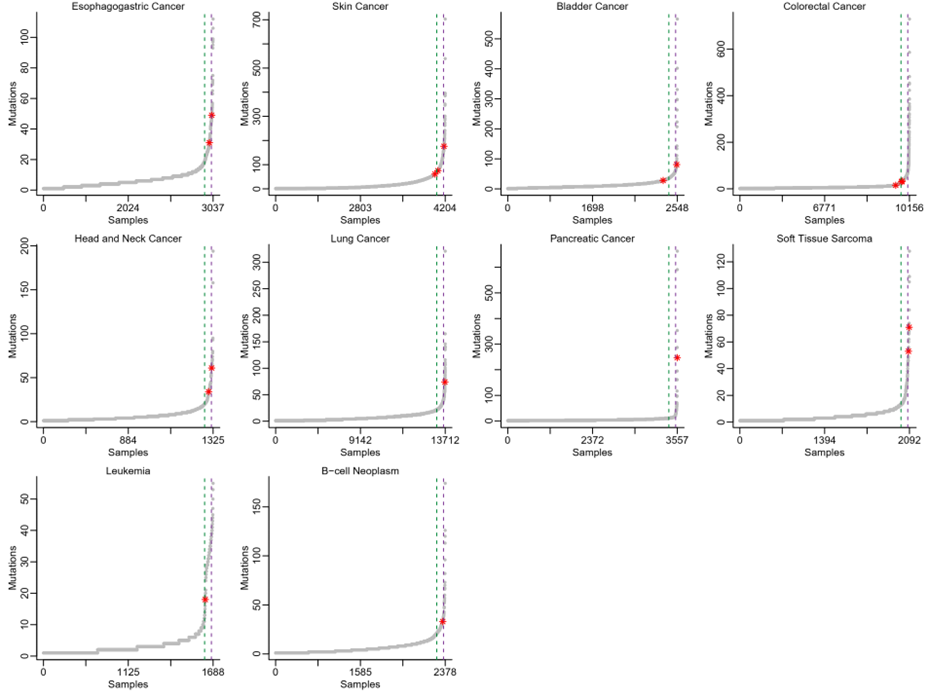
**

**Figure S5. Mutation burden of GCV positive samples relative to other samples by cancer type.** GCV positive samples are indicated by red star for each cancer type. Samples for each cancer type are ordered by the number of mutations. Top 1% and 5% are indicated by purple and green line respectively.


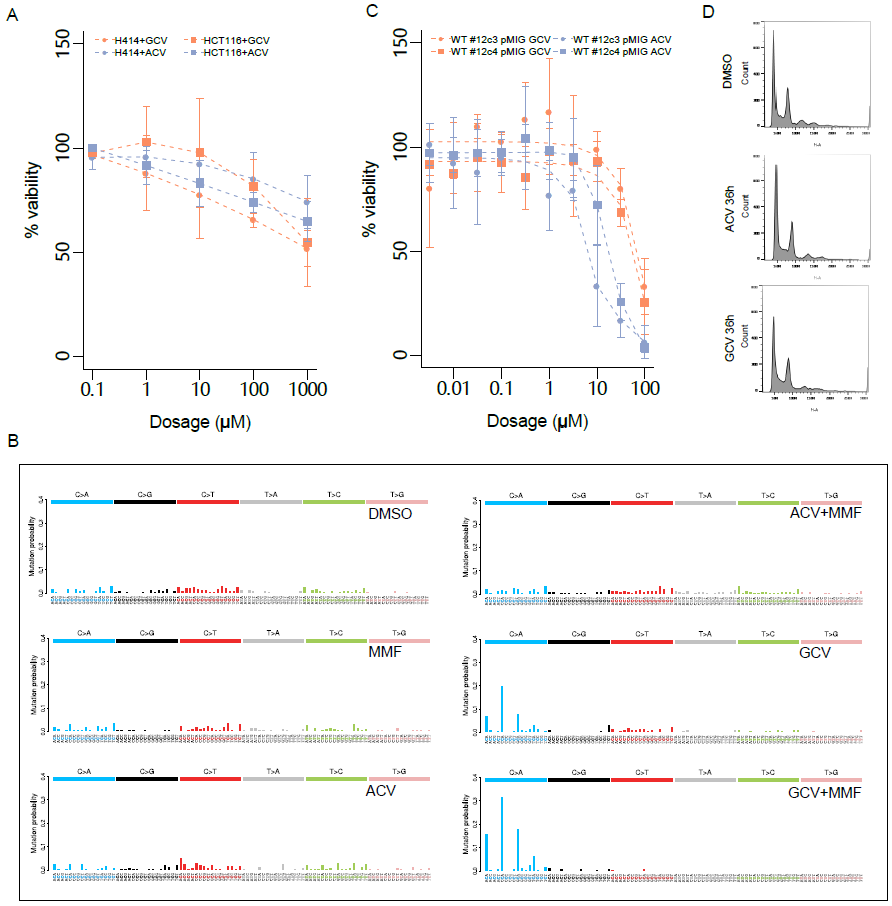


**Figure S6. Treatment of cells with ganciclovir and acyclovir.** (A) Cell viability curve of GCV and ACV treated H414 and HCT116 cells based on typan blue staining. Mean and standard deviation from 3 replicates shown. (B) Full trinucleotide mutational spectrum of DMSO, MMF, ACV, ACV+MMF, GCV and GCV+MMF treated H414 cells. Concentration of MMF, ACV and GCV is 1 µM, 100 µM and 100 µM, respectively. (C) Cell viability curve of GCV and ACV treated myeloid cells. Mean and standard deviation from n=3-4 replicates shown. (D) Flow cytometry analysis for cell cycle distribution of H414 cells with vehicle (DMSO), 100 µM ACV and 100 µM GCV after 36 h treatment.


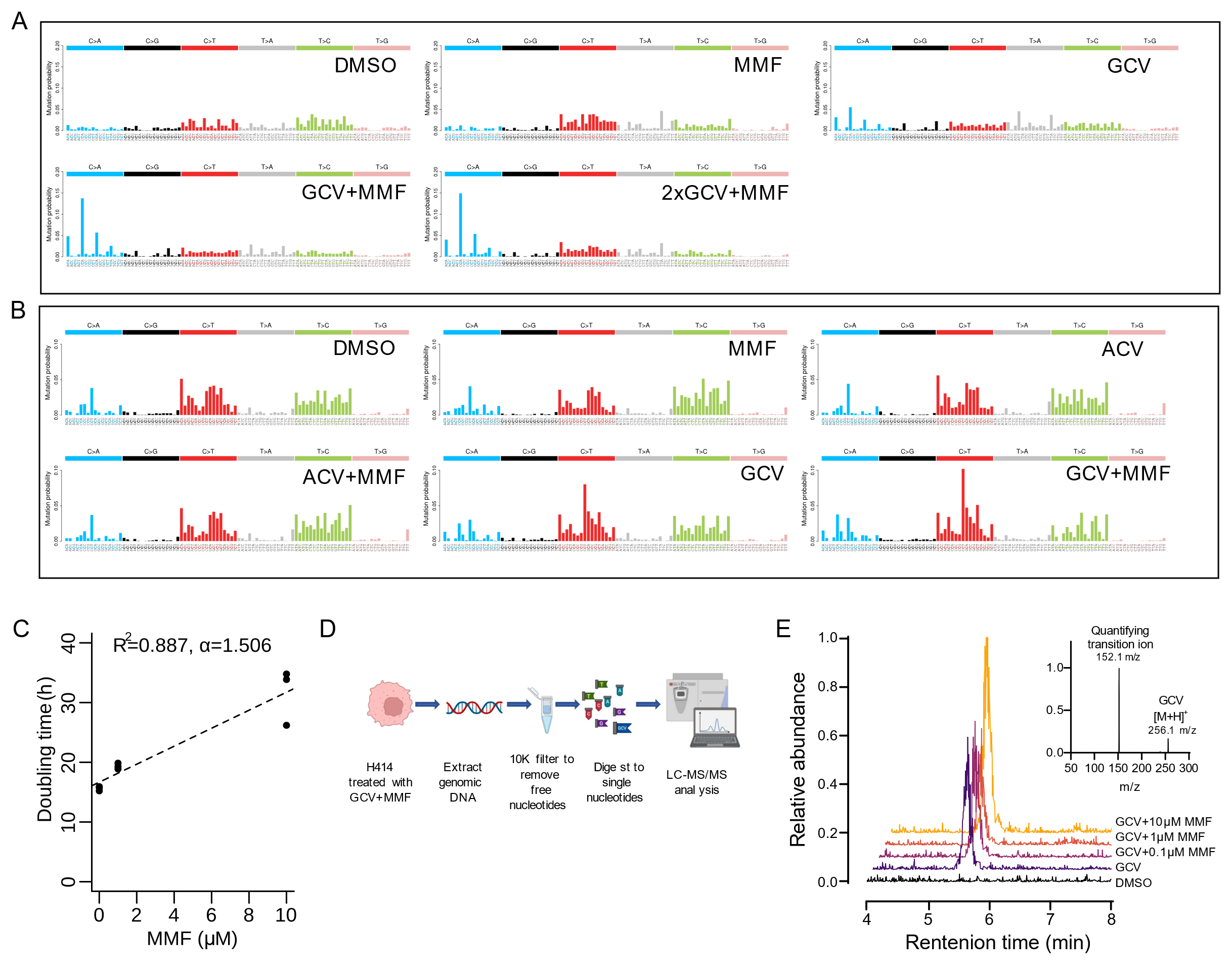


**Figure S7. Treatment of cells with ganciclovir (GCV) and mycophenolate mofetil (MMF).** Trinucleotide mutational spectra from whole genome sequencing data of H023 organoid cells (A) and HCT116 cell line (B) treated with vehicle (DMSO), GCV, MMF or combinations. For H023 the concentration of MMF is 1 µM and GCV was 20 µM and 40 µM (2x). For HCT116 the concentration of MMF is 1 µM, while ACV and GCV was 100 µM. (C) Scatterplot and linear regression of doubling time in hours versus MMF concentration. (D) Schematic of mass spectrometry-based assay for quantifying GCV incorporation in genomic DNA. (E) Chromatogram for the quantifying transition ion (152.1 m/z) from genomic DNA of H414 cells treated with (DMSO), 100 µM GCV, with increasing concentrations of MMF. The mass spectrum representing the transition (256.2 m/z 🡪152.1 m/z) is shown in the inset.

**
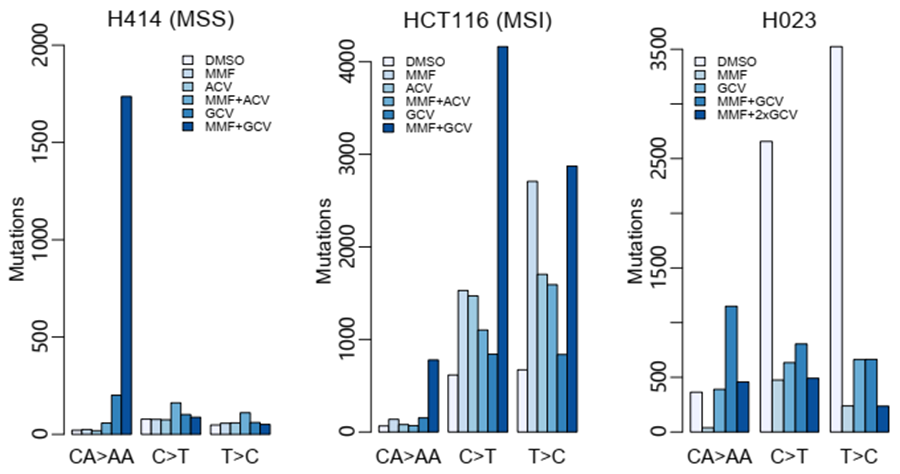
**

**Figure S8. Absolute mutations for GCV-induced mutations(CA>AA), C>T and T>C mutations across H414, HCT116 cell line and H023 organoid models.**

**
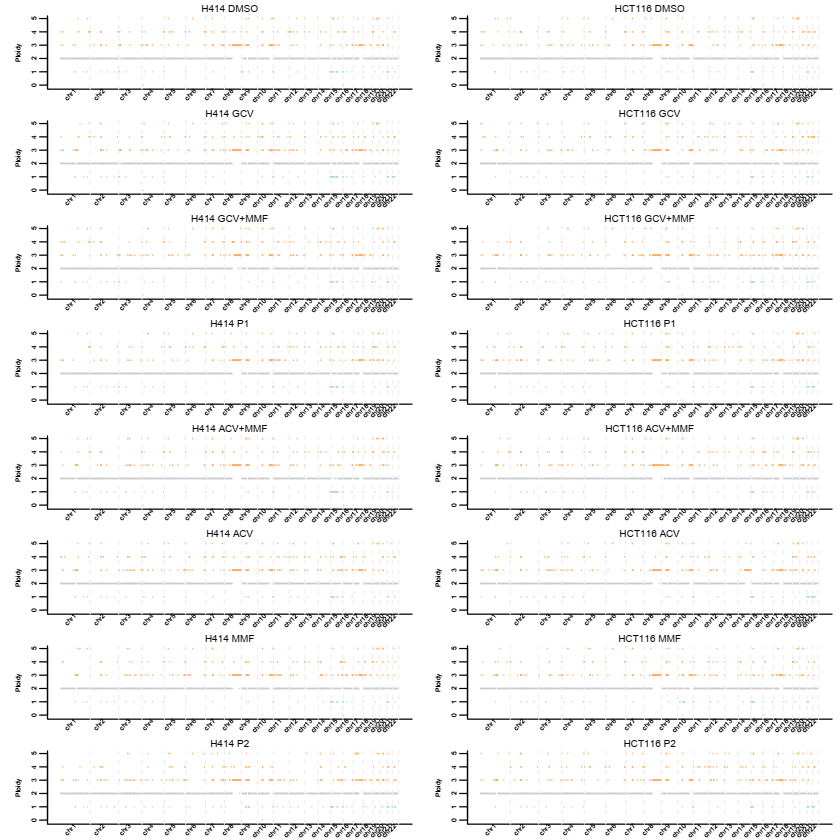
**

**Figure S9**. Copy number variation across the genomes of H414 and HCT116 clones. The ploidy values are calculated using Control-FREEC from each sample’s whole genome sequencing data.

**
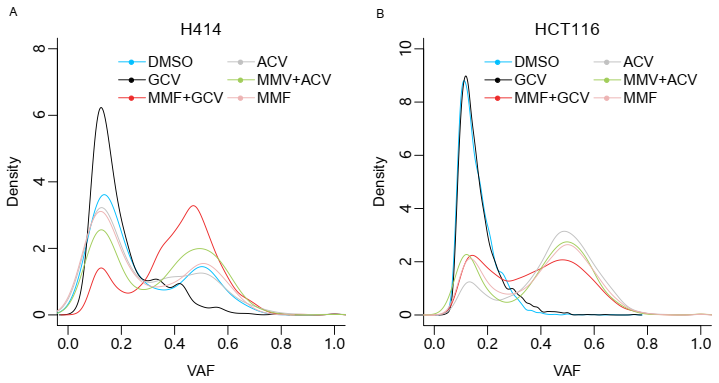
**

**Figure S10**. Histogram showing the distribution variant allele frequency (VAF) of mutations from diploid regions in each clone of H414 (A) and HCT116 (B).
